# Understanding Civility

**DOI:** 10.1007/978-981-33-6706-7_2

**Published:** 2021-03-03

**Authors:** Matteo Bonotti, Steven T. Zech

**Affiliations:** Politics and International Relations, Monash University, Melbourne, Australia

## Abstract

This chapter introduces the concept of civility and identifies its two main dimensions. The first, politeness, is related to norms of etiquette and good manners. Politeness involves both structural and agential components, and presents two important functional aspects: it can be used to signal respect and consideration for others, and it can help facilitate social cooperation. The second dimension of civility, public-mindedness, involves treating others as free and equal members of society. Civility as public-mindedness presents two sub-dimensions. The first, moral civility, demands that we respect other people’s fundamental rights, liberties, and equal civic standing, for example by avoiding racist and discriminatory speech and behaviour. The second, justificatory civility, requires that we refrain from justifying political rules based on self-interested or sectarian reasons.

## Introduction

This book examines COVID-19 through the lens of civility. A focus on civility is important for two reasons. First, the concept of civility often features prominently in public debate. Society expects people to adhere to acceptable behaviours in a range of contexts in public life. Some individuals are quick to levy accusations of incivility against those deemed to have violated these norms of behaviour. For example, when American football player Colin Kaepernick began to kneel during the national anthem as a protest against racial and social injustice, US President Donald Trump viewed the action as uncivil and disrespectful. Trump suggested: ‘[y]ou have to stand proudly for the national anthem or you shouldn’t be playing, you shouldn’t be there, maybe you shouldn’t be in the country’. He went on: ‘[t]hat's a total disrespect of our heritage. That’s a total disrespect of everything that we stand for’.^1^ Kaepernick’s actions infuriated Trump, who offered vocal support when former Vice President Mike Pence and his wife left the stadium as a counterprotest before a 2017 49ers-Colts NFL game in Indianapolis.^2^ Kaepernick faces ongoing accusations of incivility. But his case also raises an important question: should we always be civil? As Kaepernick himself more recently reasoned, ‘When civility leads to death, revolting is the only logical reaction’.^3^ Sometimes we need to be uncivil in order to highlight injustice and fight against it, especially when other means of doing so are not (or no longer) effective.^4^

In another recent high-profile US incident, the owner of a restaurant in Lexington, Virginia asked former White House press secretary Sarah Huckabee Sanders to leave the premises. Several LGBTIQ + waitstaff objected to her defence of President Trump’s discriminatory policies aimed at transgender people. The incident raised important questions regarding moral and tactical choice in an ‘age of outrage’ and contentious politics.^5^ Those who supported the action viewed the request as appropriate given the administration’s violation of moral principles of equal respect for all citizens—a key dimension of civility, as we will explain later in this chapter. However, pundits from the political right also saw the restaurateur’s action as uncivil, since asking Huckabee Sanders to leave the premises violated widely recognized politeness norms. This deterioration of civil exchange can have far-reaching effects.^6^ Both sides identified transgressions of civility norms by the other party but each focused on a distinct dimension of civility.

This leads to the second reason why it is important to focus on civility: the contested nature of the concept in the scholarly literature, particularly in political theory and philosophy. There is disagreement, first, regarding the meaning of civility (and therefore incivility). Second, even when there is agreement on the meaning of (in)civility, there may be disagreement as to whether specific instances of speech or behaviour should be considered civil or uncivil based on the agreed upon conception.

So, what is civility? Existing academic work provides different and often contrasting definitions of the concept. However, we identify two main understandings. On the one hand, civility is often associated with norms of etiquette and politeness: to be civil, in this first sense, implies to speak and act in ways that comply with these norms. On the other hand, it is linked with the idea of public-mindedness: to be civil, in this second sense, means to display a commitment to the public good, not just to one’s personal or sectarian interest, and to treating others as free and equal. This second understanding of civility is famously captured by John Rawls’s claim that we have a ‘duty of civility’ to only appeal to public reasons (i.e. reasons that our fellow citizens could understand and find persuasive) when we justify political rules.^7^ But civility as public-mindedness, we will see, can also be understood in a non-justificatory sense, requiring that we refrain from treating others in ways that are discriminatory or hateful. In this chapter, we provide an overview of the politeness and public-mindedness dimensions of civility in order to set the stage for our analysis in the subsequent chapters, which will focus on how COVID-19 challenges our ability to be civil, but also creates opportunities for people to find new ways of being civil towards one another in these challenging times.

## Civility as Politeness

Much of the scholarly literature in political theory and philosophy views civility as a virtue linked to etiquette and good manners. Derek Edyvane, for example, argues that ‘civility is bound up with the idea of what it means to be civilised, to be well-mannered or polite; its focus is on standards of behaviour in our dealings with others in everyday life’.^8^ Likewise, Cheshire Calhoun refers to ‘polite civility’, which ‘has been understood as the mark of the competent participant in the social settings of everyday life’.^9^ According to her, ‘polite civility enables the social participant to avoid barbaric and potentially disgusting bodily displays’.^10^ In line with these accounts, we view politeness as a feature of speech or action that complies with certain social norms prescribing appropriate modes of exchange and interaction.

A key feature of civility as politeness is that it is intrinsically dependent on ‘generally agreed upon, often codified, social rules’.^11^ Importantly, such rules are always contextual, since they are based on norms and customs that vary between (and within) different societies. Furthermore, these norms constantly evolve.^12^ Partly for these reasons, Strachan and Wolf observe that ‘measuring the level of civility present in society…is especially difficult because the specific behaviours defined as appropriate in one culture, or even in different settings within the same culture, can be inappropriate in others’.^13^ Likewise, in his analysis of what he calls ‘republican civility’, Daly observes that ‘the bodily and linguistic techniques that constitute civility [as politeness] are highly encoded partly because they embrace a situational or cultural specificity which will appear arbitrary and perhaps incomprehensible to those not already endowed with it’.^14^ In summary, civility as politeness is grounded in cultural and social norms that vary between contexts and may evolve over time, sometimes rather quickly.

The politeness dimension of civility involves both structural and agential elements. We will examine them in turn in the following subsections.

### Structural Elements of Politeness

The social norms of politeness constitute its structural elements. These norms are generally based on social or cultural identities, as well as on social actors’ role(s) and context, and may reflect different degrees of formality depending on the situation.

Social identity is a clear source of politeness norms. As individuals, we all occupy multiple and simultaneous identities. These include identities based on gender, race/ethnicity, class, religion, culture, and language, among others. These different identities can be sources of various politeness norms. For example, a person’s religious faith may contain norms of politeness concerning how one should relate to or greet other individuals (both co-religionists and not) or how one should dress when entering a place of worship.^15^ Some of these norms may interact with gender dimensions of identity, establishing more specific guidelines on how one should relate to people of the opposite sex or how men and women should dress in public.^16^ And gender itself can be tied to politeness norms independently of religion.^17^ Language may also be a source of politeness norms, by providing its speakers with socially legible ways (e.g. through its lexicon, grammar, idiomatic expressions, or even the tone and pitch in the way we speak) of expressing politeness.^18^

Norms of politeness also arise from the many social roles that we occupy, regardless of whether they are connected to specific social identities. For example, particular norms often guide our interactions with family and friends. Those norms may differ from those of other families or other groups of friends, for example with regard to eating times and etiquette, or the way we make interpersonal requests.^19^ Similarly, norms of politeness also exist in the workplace. Co-workers greet each other on a daily basis following specific norms, and may adhere to politeness norms that regulate the use of a shared kitchen or a shared printer. Norms of politeness can regulate interactions between employers and employees, in ways that may reflect the hierarchical relationship between them.^20^ Furthermore, politeness norms may influence forms of participation in political and social movements^21^ and can structure the relationship between leaders and followers.^22^ For example, followers may be expected to bow or not turn their back to leaders in public/official settings and those same leaders must abide by norms of politeness to maintain status and respect. This may involve restraint in speech or behaviour, acknowledging and greeting their followers, and more generally maintaining the so-called ‘dignity of the office’.^23^

In addition to social identity and roles, the context of politeness matters too. In the above situations, different factors affect how much, when, and where different social norms apply. Context involves, first of all, power and authority relations. These clearly affect the intensity with which different social norms apply in asymmetrical contexts, such as the aforementioned workplace context and leader/follower relationships.^24^ The latter may affect the predominant direction and quality of politeness norms, regulating the behaviour of those in subordinate positions more (or, at least, in different ways) than in the case of those who occupy dominant positions.^25^ In summary, power and authority affect the form of, and compliance with, social norms.

Politeness is also linked to environmental considerations. For example, since politeness is often understood as regulating social distance (as well as psychological distance), spatial considerations are relevant,^26^ especially in public spaces and urban environments.^27^ In this sense, different norms of politeness may often apply in physical as opposed to digital spaces. Moreover, even though norms of politeness often mandate maintaining a certain distance from other people in public spaces (unless other constraining factors—e.g. a crowded bus—prevent one from complying with them), maintaining too much distance from others may sometimes also be perceived as impolite, as when someone decides to sit in the last row of an empty seminar room during a talk.

Furthermore, one might speculate that certain features of our environments can constitute an affront to senses (e.g. loud noises, unwelcome sights, or bad smells), something that Joel Feinberg famously labels ‘offensive nuisances’.^28^ This seems to suggest that politeness norms may also be tied to the design and form of that environment, and that certain buildings, materials, and colours (and not only the people who act within them) could sometimes be perceived as offensive or impolite. And, indeed, Welsh architect Trystan Edwards once famously claimed that we could distinguish between ‘rude’ and ‘polite’ buildings.^29^ Take, for example, the so-called ‘Walkie Talkie Building’ in London, which has been criticized for reflecting intense sunlight off its glass windows.^30^ Likewise, the use of certain colours in the visual environment might be perceived as more or less impolite in different contexts depending on the relevant social and cultural norms.^31^

Digital spaces may also involve different degrees of spatial proximity. For example, recent research has shown that the kind of online forum through which people interact creates higher or lower levels of virtual spatial proximity among users, resulting in different levels of politeness and impoliteness. More specifically, forums to which users contribute anonymously and on a one-off basis create a greater virtual spatial distance between them, resulting in higher levels of impoliteness. Conversely, forums in which users engage in more individual one-to-one iterated interactions tend to result in higher levels of politeness.^32^

Norms of politeness also vary in their degree of formality and informality. Most politeness norms tend to be informal, and violations are only accompanied by social sanctions. Think, for example, of norms concerning the practice of gift giving at Christmas.^33^ In other cases, politeness norms may become codified via law and rigid etiquette (or protocols within organizational contexts). Consider, for instance, norms of etiquette preventing people from accessing certain venues unless they wear a suit and tie, or laws that make swearing illegal.^34^ In some cases, informal or non-legal social norms may gradually acquire legal status, as when norms that attach impoliteness to certain behaviours in relation to sacred places become legally enforced. Moreover, norms of politeness may include norms of display and norms of concealment, where one can be polite either by saying/doing something or by refraining from doing so.^35^

Finally, we should consider two additional dimensions of politeness norms. First, so far we have focused on norms that are linked to specific social identities and roles, and which are in many ways contextual. There is general consensus around the view that politeness norms are socially and culturally specific.^36^ However, one might argue that at least some politeness norms have a (nearly-)universal scope. Some scholars, for example, have pointed to a universal desire for not ‘losing face’ as evidence of the presence of universal norms of politeness.^37^ Furthermore, one might also argue that some of the aforementioned affronts to the senses^38^ may also constitute instances of impoliteness across different social and cultural contexts, perhaps because they are generally types of offenses not mediated via culturally specific beliefs. Second, politeness norms are not static.^39^ The structural factors that we examined in this section are not fixed or rigid—politeness norms continually evolve. The actors involved, whether in a family, a workplace, or a broader political community, continually (re)negotiate those norms. The next section focuses on these agential dimensions of politeness.

### Agential Elements of Politeness

In addition to the structural elements of politeness—i.e. the sources of social norms that define what it means to be polite—scholars must recognize the role of agency in acting politely. Not only do norms of politeness change and evolve, but individuals also need to navigate such norms. As one scholar observes, civility [as politeness] is the ‘practical ability of individuals to distinguish between different social roles and contexts and to differentiate their behaviour accordingly…[which] crucially involves an element of judgment.’^40^ So, how is this practical ability or judgment exercised?

Agents need to exercise a degree of critical engagement in order to be polite. Humans learn what norms of politeness are and subsequent practices usually reinforce expected behaviours.^41^ Under normal, everyday circumstances, politeness norms are reasonably clear. People mostly understand the demands of a situation and how to interact with others. Decisions about how to comport oneself on public transportation^42^ or how to greet a stranger can be routine in familiar contexts and require minimal reflection.^43^ Yet, some situations prove more challenging to navigate. For example, travelling to culturally distinct tourist destinations or attending an unfamiliar religious site during an interfaith wedding ceremony may introduce moments of uncertainty regarding polite behaviour. The uncertainty and challenges can become even more pronounced in moments of crisis that disrupt social life in unprecedented ways—the COVID-19 pandemic is one of those moments.

Critical engagement requires recognising and learning new politeness norms, as well as how they apply when we interact with others across a range of contexts. This involves several dimensions. First, people need to undertake information-seeking actions that include identifying, studying, and questioning new behaviours. They also need to observe and interpret the actions or others. Furthermore, critical engagement involves self-awareness and the ability to identify and rationally assess one’s own behaviour and how it relates to old and new expectations. Novel circumstances demand that individuals reflect upon new conditions before they take action, rather than simply relying on disposition or habitus.^44^

Second, people need to become aware of and overcome additional cognitive limitations and information access issues. For example, motivational and confirmation biases, framing effects, and information processing capabilities may present obstacles to learning and adaptation in these environments.^45^ Access issues related to information infrastructure, technology, and language issues—as well as imperfect information or overt attempts to mislead the public—could all complicate the transmission and adoption of new politeness norms. Situational tendencies related to risk assessment further complicate decision-making and behaviour.^46^

Third, an absence of scientific consensus regarding which information is accurate and appropriate may inhibit the adoption of and adherence to some politeness norms. People internalize and observe a wide range of normative social behaviours, and science may support or refute the assumptions behind those behaviours. For example, party etiquette might ask that attendees refrain from ‘double-dipping’ a chip into salsa after taking a bite, and we are encouraged not to eat food that has fallen on the ground based on a ‘five-second rule’. However, it seems that research findings confirm the social harms only for the former scenario,^47^ but not for the latter.^48^

Finally, other people and institutional structures may affect the way in which people navigate norms. This might take the form of political leaders intentionally passing along biased or distorted information based on politicized or self-interested agendas. For example, the prominent Italian politician Matteo Salvini, as well as some Italian right-wing media, often accuse their critics of being impolite.^49^ This is problematic since the ability to discern when norms of politeness ought to be complied with and when they might be breached should be central to our critical engagement with such norms. Indeed, in some cases incivility expressed as impoliteness can be used as a tool for signalling and contesting unjust policies or institutions.^50^ Public accusations of impoliteness, while technically correct (e.g. Salvini’s critics may well be impolite when they swear at him), might delegitimize some people’s use of impolite speech or behaviour in ways that prevent the audience from critically assessing their appropriateness in certain circumstances (e.g. protesting against restrictive policies targeting migrants, such as those Salvini himself endorses).

Various forms of censorship could prevent people from learning about relevant politeness norms as well. Those who lack exposure to norms of polite behaviour or speech, for instance, could (un)intentionally stigmatize others. Education levels, generational gaps, and class divides could hinder people’s ability to identify and fully understand the content and meaning of different norms of politeness. For example, older white residents in the American South may not observe shifts in politically correct racial references to blacks.^51^

Overcoming all these obstacles involves individual costs, both financial and in terms of time and effort. Learning new things when filling a new role or in the context of cultural difference may require significant investment in time and information-seeking activities. Intercultural competencies are crucial in many settings, including tourism, where a traveller or volunteer may need to acquire translation dictionaries or take short language courses in preparation for a visit.^52^ Alternatively, the investment in training for scenarios like international disaster deployment^53^ or relationship-building during counterinsurgency campaigns^54^ can have much higher stakes. There are also various kinds of social and cultural costs. These may involve ‘individual compromising’ if certain actors temporarily give up preferred norms to comply with those that apply to the relevant situation they find themselves in. Individuals might also incur social and political opportunity costs associated with various forms of in-group stigma and loss of legitimacy or authority within their group. For example, some former white supremacists are often stigmatized by other extremists when they attempt to politely engage with members of minority groups they used to target.^55^

### The Functionality of Politeness: Signalling and Social Cooperation

The politeness dimension of civility serves an important function in social and political life. Acts of politeness can be used as a signal. For example, for Edyvane, civility as politeness represents a mode of interaction that displays or communicates a recognition of ‘one another as people among whom [we] must live’,^56^ through compliance with a particular set of social norms. Furthermore, some expressions of politeness norms can move beyond simple recognition and communicate respect to others and acknowledge their dignity.^57^ For example, Calhoun argues that politeness ‘involves a display of respect, toleration, or considerateness’.^58^ Central to Calhoun’s conception of civility is the idea that ‘civility…is an essentially *communicative* form of moral conduct’.^59^

The signalling function of politeness is important for several reasons. While there are obvious implications for economic exchange, we focus on the social and political aspects. Politeness norms facilitate and reinforce social cooperation in the presence of disagreement—they allow for and promote a healthy and well-functioning society. Sune Lægaard actually describes this aspect as the primary function of civility; it has the pragmatic role ‘to ease social tensions in order to facilitate social interaction and collaboration across differences and the resulting disagreements’.^60^ Civility as politeness essentially acts as a ‘lubricant’ for social cooperation.^61^

The politeness dimension of civility affects social interactions and behaviours across a range of sites including the home,^62^ the workplace,^63^ educational settings,^64^ and public places in the community.^65^ Politeness also influences interactions in familial, friendship, romantic, and business relationships. For example, (im)politeness in the workplace can have significant and far-reaching effects on important outcomes like team cohesion,^66^ as well as employee productivity.^67^ Furthermore, polite behaviours can reinforce cooperative relationships in an office setting, while incidents of impoliteness can harm prospects for ongoing productive exchange.^68^ Overtly rude behaviours have also been shown to reduce performance levels and helpfulness in experimental settings.^69^

There are also implications for (im)politeness in the political realm. Politeness can have direct effects on outcomes related to specific election debates,^70^ along with how the public reacts to politicians' behaviours in electoral politics.^71^ Perceptions about even ‘minor incivilities’ can have far-reaching effects on public perceptions and fears, as well as subsequent policies.^72^ Impolite political discourse can have lasting effects on matters of decorum that help to structure party politics, and a political atmosphere of ongoing impoliteness can further reinforce entrenched political positions.^73^ However, an insistence on polite behaviour could also stifle acts of dissent aimed at unjust policies that threaten desirable political ideals.^74^ We should also recognize that political interactions can take place in public or private spheres and in physical or virtual spaces.^75^

Politeness is also important for diplomatic relations,^76^ as well as for the efficacy of foreign policy^77^ and prospects for international cooperation more broadly. How states treat migrants and asylum-seekers during processing may affect their international and domestic reputation. For example, polite or impolite behaviour by officials at border checkpoints or during processing can provide an indicator as to whether a state lives up to the fair assessment of applicants and to practices expected from liberal democratic countries.^78^ In the realm of contentious politics, the form that civil resistance movements take on, including how they may or may not comply with social expectations regarding strategy, influences the likelihood of their success and failure.^79^ Even in extreme circumstances like violent armed conflict, civilians can create institutional mechanisms to encourage polite and respectful behaviour by insurgent and counterinsurgent forces.^80^

The COVID-19 crisis has disrupted politeness norms across the globe in economic, social, and political spaces. These disruptions will undoubtedly have implications for how people and states interact with each other. When politeness norms are uncertain or changing, as during the current pandemic, it is more difficult for people to comply with them. This will in turn also affect their ability to use polite speech or behaviour to effectively signal their commitment to social interaction and cooperation, thus potentially creating or exacerbating social tensions. We will return to these points in the next chapter.

## Civility as Public-Mindedness

Politeness, especially when understood as a communicative virtue, constitutes an important dimension of civility, as we explained in the previous section. However, it is not the only dimension. Several scholars understand civility mainly as a political virtue, specifically a civic virtue that is inherently related to liberal politics.^81^ The political dimension of civility emphasizes our responsibilities and duties as *citizens* of a liberal democracy, rather than simply as individuals interacting in different everyday social settings. Edyvane, for example, points out that this political understanding of civility is ‘bound up with the idea of an association of citizens, and includes cognate ideas of the civic, the civil and the civilian; it concerns one’s status and duties as a member of a political community, as a citizen with certain rights and responsibilities’.^82^ Central to this understanding of civility is compliance with fundamental liberal democratic values, as well as a commitment to the common good rather than to personal or sectarian interests. To be civil in this political sense means to engage in ‘a *kind* of politics, a *type* of political discourse that does not harm, injure, or offend fellow citizens’.^83^ Doing so can contribute to better democratic governance and social coexistence in the long term.

While this second dimension of civility is often referred to as ‘political civility’,^84^ we prefer the term *civility as public-mindedness*. The label ‘political civility’ might inadvertently suggest that this kind of civility is solely relevant to limited aspects of the political realm like institutions, political parties, or electoral campaigns. Civility as public-mindedness has a broader meaning, and involves recognizing others as free and equal members of society. According to Richard Boyd, for example, civility involves ‘the mutual recognition of others as our moral equals’.^85^ Likewise, Robert Pippin claims that civility entails acknowledging others’ ‘equal status as free agents within a cooperative enterprise’.^86^ However, it is important to distinguish between two different sub-dimensions of civility as public-mindedness. While these two aspects overlap, and both involve treating others as free and equal citizens, each of them points towards a specific dimension of public-mindedness and specific duties associated with it.

### Moral Civility

The first dimension of civility as public-mindedness refers to the way we interact with others via our actions and speech in societies characterized by diversity and disagreement. We call this *moral civility*. According to this dimension, to respect others as free and equal means to respect their fundamental rights, liberties, and equal civic standing. It means, for example, refraining from using physical violence against others,^87^ discriminating against them,^88^ or using racist or other types of expression that portray members of certain groups as physically, intellectually, or morally inferior. When it comes to the latter point, perhaps the most prominent defence of moral civility in recent years is provided by Jeremy Waldron in his book *The Harm in Hate Speech*.^89^ As Teresa Bejan points out, ‘[f]or Waldron…[s]ome speech about others and their fundamental identities and commitments is simply *so* uncivil, so intolerant, degrading, and disrespectful that it constitutes a form of *persecution* against which a tolerant society can and should act’.^90^ Here two clarifications are required.

First, while apparently overlapping, civility as politeness and moral civility are not the same thing. In some cases, these two dimensions of civility can be tightly connected. As we mentioned earlier, civility as politeness can be used to communicate respect to others and acknowledge their dignity, two key aspects of moral civility. According to Sarah Buss, for example, ‘by behaving politely we are, in effect, “saying” something to one another…[and]… acknowledge[ing] one another’s special dignity’.^91^ Yet, in other cases, politeness and moral civility may come into tension, as when polite means are used to advance morally uncivil causes, or impolite behaviour or speech is employed to advance morally civil goals.^92^ For example, ‘polite Nazis’ may abide by norms of good manners and etiquette for purely strategic reasons, while advancing political goals that are morally uncivil since they deny a free and equal status to members of certain groups.^93^ Conversely, the use of impolite behaviour or speech can signal dissent towards unjust policies or institutions to help advance morally civil goals.^94^ Second, while moral civility is often tied to liberal democratic institutions, this is not always the case. For example, while all liberal democracies have laws against physical violence, not all of them have laws regulating racist or hate speech, at least not in the same way. Where (most) racist speech is not regulated (e.g. in the US), it can be argued that citizens have the legal right, sanctioned by liberal democratic institutions (e.g. the US First Amendment), to violate moral civility with particular speech acts.

### Justificatory Civility

The second dimension of civility as public-mindedness concerns the public justification of political rules in liberal democracies. According to this view, in societies characterized by reasonable pluralism and disagreement, citizens and public officials treat each other as free and equal if they justify political rules by appealing only to public reasons. These are reasons that all of them could in principle understand and accept, rather than reasons grounded in their different worldviews, identities, and beliefs. We call this *justificatory civility*. The most influential account of justificatory civility is perhaps the one provided by John Rawls, for whom the duty to appeal solely to public reasons during the process of public justification is a ‘duty of civility’.^95^ Complying with this duty means renouncing the use of religious or other controversial arguments when justifying political rules, thus engaging in ‘reasonable public discourse’.^96^

Here we should draw a distinction between two different components of justificatory civility. The first consists of fundamental political values that are widely shared in liberal democratic societies, and include for example civil and political rights and liberties, equality of opportunity, and values linked to the promotion of the common good.^97^ These constitute the *moral content* of justificatory civility. It is important to stress that justificatory civility demands not only that we appeal to these values, rather than to sectarian or controversial ones, during the process of public justification; it also requires that we balance them in reasonable ways. To do this, we need to provide reasons that ‘represent a plausible balance of political values. An argument, even if based on a political and free-standing value, fails to be a reasonable public justification if it does not plausibly address other political values that may be at stake’.^98^ The second component of justificatory civility includes what Rawls refers to as ‘guidelines of inquiry: principles of reasoning and rules of evidence in the light of which citizens are to decide whether substantive principles properly apply and to identify laws and policies that best satisfy them’, as well as ‘the methods and conclusions of science when these are not controversial’.^99^ These form the *epistemic content* of justificatory civility.

Accounts of justificatory civility differ in many respects. There is disagreement, for example, as to whether the duty of civility only applies to constitutional matters or also more generally to all legislation.^100^ Furthermore, while some only assign that duty to legislators and public officials,^101^ others extend it to citizens more broadly.^102^ More importantly, there are different accounts of the structure of public reason. Some, following Rawls, endorse a consensus approach to public reason and argue that public reasons (or, at least, the premises and standards on which public reasons are grounded) should be shared among all (idealized) members of the public.^103^ Others reject this position and defend convergence accounts of public reason where justification does not require shared reasons or premises but only that political rules be justified to each and every citizen based on reasons that they will find persuasive from the perspective of their own beliefs and values.^104^ Convergence accounts of public reason therefore are in tension with the Rawlsian duty of civility. The latter demands that we refrain from appealing to religious or other controversial values during the process of public justification, but such appeals may be permissible under convergence accounts if they help citizens or legislators to justify political rules to those who endorse those views, e.g. members of certain religious groups.^105^ This does not imply that one is uncivil (in the justificatory sense) if they endorse a convergence account of public justification. However, civility grounded in the convergence view will have different implications for public justification: to be civil in this sense will mean to provide each of our fellow citizens with reasons in support of political rules that they can find acceptable from their own perspective, rather than striving to articulate widely shared reasons that all our fellow citizens may accept.

A final aspect should be considered. The duty of civility concerns the public justification of political rules, which is tied to their aims and goals, rather than their outcomes and consequences. For this reason it is often assumed that as long as laws and policies are publicly justified (based on whichever conception of public justification one endorses), the fact that they might end up favouring or burdening certain citizens more than others is not morally relevant from the perspective of public justification. However, this constitutes an incorrect, or at least incomplete, account of public reason and public justification. The outcomes and consequences of political rules are and *should* be relevant to public justification. Based on Rawls’s idea of the ‘strains of commitment’,^106^ some scholars point out that it would be unreasonable to implement political rules (even if in principle justified based on public reasons) that would impose intolerable strains upon certain citizens.^107^ For example, members of religious groups could not reasonably be expected to accept laws that would make it overly difficult for them to pursue their religious beliefs and practices, even if these laws in principle aim to realize public-minded goals.

In this book, our analysis of justificatory civility is based on a number of premises, which we assume without further defence. First, we contend that justificatory civility concerns all political rules and not only those concerning constitutional matters; therefore all legislation related to COVID-19 ought to be publicly justified. Furthermore, we postulate that when it comes to the public justification of policies, the Rawlsian duty of civility applies solely to those who occupy decision-making positions, like politicians and public officials. We also share the belief that consensus accounts of public reason are more desirable than convergence ones, and that justificatory civility should take into account the outcomes and consequences of political rules that result in strains of commitment for some individuals and groups within society.

At this point it is important to acknowledge that both moral and justificatory civility, as we presented them in this chapter, may be contested by some. For a start, only those liberals who, like Rawls, are committed to political liberalism and public reason, will be prepared to consider justificatory civility a key dimension of civility as public-mindedness (or of civility *tout court*). Liberal perfectionists who criticize the ideals of public reason and public justification,^108^ for example, might embrace moral civility but reject justificatory civility. Furthermore, some may consider moral civility itself too demanding. In her book *Mere Civility*, for example, Teresa Bejan rejects Rawls’s justificatory civility and criticizes Waldron’s moral civility. More specifically, she argues.

[f]ollowing Rawls, some theorists invoke civility in arguments about public reason and other constraints on public deliberation, while others, like Waldron…, stress the negative effects of uncivil speech on diversity and dignity for members of tolerant societies…[T]hese theorists’ robust understandings of civility often do create covert demands for conformity that threaten to civilize disagreement by putting an end to it entirely…[T]he ‘uncivil’ are left only two options: a sincere conversion to the fundamentals of political liberalism or silence.^109^

Against the Waldronian and Rawlsian views of civility, which roughly correspond to what we call moral and justificatory civility, Bejan defends the idea of ‘mere civility’ advocated by the Puritan exile and dissenter Roger Williams, who founded the colony of Rhode Island. Mere civility involves ‘a minimal adherence to culturally contingent rules of respectful behavior compatible with, and occasionally expressive of, contempt for others and their beliefs’.^110^ We stipulate, without further argument, that Bejan’s conception of civility is too narrow to most effectively engage many of the social and political challenges posed by COVID-19. We contend that our more expansive view of civility as public-mindedness, with its moral and justificatory dimensions, offers a richer framework for understanding those challenges.

## From Civility to Incivility

Based on the foregoing analysis of the different dimensions of civility, we identify a number of ways in which incivility can manifest in liberal democracies. Each of these dimensions of incivility will be relevant to our analysis in Chapters 10.1007/978-981-33-6706-7_3 and 10.1007/978-981-33-6706-7_4, where we illustrate how COVID-19 has resulted in a loss of civility across a number of areas and settings, and what responses have been or could be adopted to recover civility in the current pandemic and during future crises.

First, incivility can manifest as a failure to comply with norms of politeness. Swearing, not greeting one’s co-workers, failing to say ‘please’ or ‘thank you’, are just some examples of how individuals might be impolite under this dimension. However, simply identifying instances of incivility as impoliteness is not sufficient. Based on our earlier analysis, it is also important to understand why people behave impolitely, and what implications this might have for their social coexistence, especially in times of crisis. Answering the former question requires delving into the structural and agential factors that may prevent individuals from abiding by norms of politeness and etiquette, especially when those norms may become uncertain, as during a crisis. Addressing the latter question entails instead examining the implications of impolite speech and action for social interaction and cooperation. We will consider both aspects in Chapter 10.1007/978-981-33-6706-7_3 in relation to the current pandemic.

Second, incivility may manifest itself as moral incivility. This may involve speech or behaviour that fails to respect other citizens as free and equal, by denying or violating their individual rights, liberties, and/or equal civic status. Physically assaulting other people, destroying property, or addressing members of certain groups with discriminatory and hateful speech, are clear instances of moral incivility. According to Jeremy Waldron, for example, hate speech denies the civic dignity and equal social status of those it targets.^111^ This, as already pointed out by Bejan, seems to be a clear instance of moral incivility. Here, however, it is important to distinguish between moral incivility and incivility as impoliteness. The latter, unlike the former, only involves a violation of social norms of politeness and etiquette but not an attack on other individuals’ free and equal status, or an infringement on their rights and liberties. Of course, what starts as merely impolite speech or behaviour (e.g. swearing) may sometimes escalate into moral incivility (e.g. hate speech and physical violence), but this shift is not inevitable and it is important to keep the two dimensions of incivility separate for analytical purposes.

Third, incivility can take a justificatory form. To be uncivil, under this dimension, means to fail to comply with the Rawlsian duty of civility, which requires appealing to public reasons when publicly justifying political rules. Here, we need to disaggregate justificatory incivility into two subtypes, based on the two elements of the content of public reason (moral and epistemic) that we illustrated in the previous section.

On the one hand, justificatory incivility may involve a failure to recognize or appeal to the moral content of public reason during the process of public justification. For example, one might appeal to reasons that overtly or covertly deny that certain citizens are entitled to basic rights and liberties. This instance of justificatory incivility clearly presents points of connection with moral incivility. After all, a racist politician who verbally abuses or physically assaults members of certain ethnic minority groups, thus being morally uncivil, is also likely to violate justificatory civility when defending or opposing certain policies and laws. Yet, the two sub-dimensions of incivility as non-public-mindedness should be kept separate. One (moral incivility) concerns how we speak to and act towards our fellow citizens in our everyday interactions. The other (justificatory incivility) relates solely to the process of public justification, which is a distinct dimension of public and political life. But justificatory incivility concerning the moral content of public reason does not manifest itself only through a failure to recognize others’ rights and liberties. It also, and perhaps more often, occurs when policymakers appeal to reasons which, while not denying others’ basic rights and liberties, are rooted in one’s self-interest or in the interest of their family, narrow social group, or partisan cause, rather than a commitment to the common good. For example, defending a tax cut based solely on the argument that it will advantage one’s party and the businesses that support it (rather than, say, contribute to economic growth for the entire society) constitutes an instance of justificatory incivility. Relatedly, one can violate justificatory civility by appealing solely to reasons grounded in controversial religious, ethical, or philosophical doctrines (e.g. Catholicism, Islam, or Millian liberalism) to justify political rules. Furthermore, politicians and public officials are also uncivil in the justificatory sense when they overly prioritize certain political values and fail to address others, or when they neglect the strains of commitment that may result from certain policies.

On the other hand, the content of public reason also involves an epistemic dimension. From this perspective, employing flawed guidelines of inquiry, or relying on controversial scientific arguments and/or methods, may result in publicly unjustified political rules. Instances of this kind of justificatory incivility may include, for example, the use of conspiracy theories to justify or oppose certain laws or policies.^112^ Likewise, one might display justificatory incivility by appealing to flawed or incomplete scientific evidence, flawed methods, and/or flawed chains of causation. Another instance of justificatory incivility related to the epistemic dimension of public reason involves the selective use of non-flawed scientific evidence for non-public-minded goals. Scientists often disagree with each other even if there are no inherent flaws in their methods or conclusions.^113^ In such cases, a policymaker might be guilty of justificatory incivility if they deliberately select (e.g. for ideological or partisan reasons) specific scientific methods and conclusions (even if not inherently flawed) to support their preferred policies, while failing to acknowledge alternative ones.

## Conclusion

In this chapter we provided an account of civility that distinguishes between its politeness and public-mindedness dimensions. With regard to civility as politeness, we illustrated its structural and agential elements, as well its functionality. When examining civility as public-mindedness, we focused instead on the distinction between its moral and justificatory sub-dimensions. This allowed us to articulate different types of incivility, setting the stage for our analysis of COVID-19 in the next two chapters. More specifically, in Chapter 10.1007/978-981-33-6706-7_3 we will examine the many ways in which COVID-19 poses unprecedented challenges to people’s ability to be polite, and what this might imply for social coexistence and cooperation, both in the short and long term. In Chapter 10.1007/978-981-33-6706-7_4 we will shift our focus to civility as public-mindedness and analyse instances of moral and justificatory incivility during the pandemic. Throughout both chapters we will also consider various measures that have been or could be implemented to counter greater incivility along these different dimensions.

### Notes

1. Anonymous, ‘Trump: NFL kneelers ‘Maybe Shouldn’t Be in Country’’, *BBC News*, 24 May 2020. https://www.bbc.com/news/world-us-canada-44232979.
2. Donald J. Trump (realDonaldTrump), 9 October 2017. Tweet. https://twitter.com/realdonaldtrump/status/917091286607433728.
3. Colin Kaepernick (Kaepernick7), 29 May 2020. Tweet. https://twitter.com/Kaepernick7/status/1266046129906552832.
4. Candice Delmas, *A Duty to Resist: When Disobedience Should Be Uncivil* (Oxford: Oxford University Press, 2018); Derek Edyvane, ‘Incivility as Dissent’, *Political Studies*, Vol. 68, No. 1 (2020): 93–109. https://doi.org/10.1177/0032321719831983.
5. David Smith, ‘How the Red Hen Affair Broke America’s Civility Wars Wide Open’, *The Guardian*, 28 June 2018. https://www.theguardian.com/us-news/2018/jun/27/red-hen-restaurant-virginia-sarah-sanders-civility-wars-trump.
6. Emily Sydnor, ‘Does Incivility Hurt Democracy? Here’s What Political Science Can Tell Us’, *The Washington Post*, 27 June 2018. https://www.washingtonpost.com/news/monkey-cage/wp/2018/06/27/does-incivility-hurt-democracy-heres-what-political-science-can-tell-us/.
7. John Rawls, *Political Liberalism* (expanded edition) (New York: Columbia University Press, 2005).
8. Derek Edyvane, ‘The Passion for Civility’, *Political Studies Review*, Vol. 15, No. 3 (2017): 345. https://doi.org/10.1177/1478929915611919.
9. Cheshire Calhoun, ‘The Virtue of Civility’, *Philosophy and Public Affairs*, Vol. 29, No. 3 (2000): 257.
10. Calhoun, ‘The Virtue of Civility’, 257.
11. Calhoun, ‘The Virtue of Civility’, 260.
12. John Kekes, ‘Civility and Society’, *History of Philosophy Quarterly*, Vol. 1, No. 4 (1984): 429–443. https://www.jstor.org/stable/27743700; Richard C. Sinopoli, ‘Thick-Skinned Liberalism: Redefining Civility’, *American Political Science Review*, Vol. 89, No. 3 (1995): 612–620. https://doi.org/10.2307/2082977.
13. J. Cherie Strachan and Michael R. Wolf, ‘Political Civility: Introduction to Political Civility’, *PS: Political Science & Politics*, Vol. 45, No. 3 (2012): 402. https://dx.doi.org/10.1017/S1049096512000455.
14. Eoin Daly, ‘Ostentation and Republican Civility: Notes From the French Face-veiling Debates’, *European Journal of Political Theory*, Vol. 14, No. 3 (2015): 313. https://doi.org/10.1177/1474885114549265.
15. Abdelaziz Bouchara, ‘The Role of Religion in Shaping Politeness in Moroccan Arabic: The Case of the Speech Act of Greeting and Its Place in Intercultural Understanding and Misunderstanding’, *Journal of Politeness Research*, Vol. 11, No. 1 (2015): 71–98. https://doi.org/10.1515/pr-2015-0004; Abdelaziz Bouchara and Bouchra Qorchi, *The Role of Religion in Shaping Politeness During Greeting Encounters in Arabic: A Matter of Conflict or Understanding* (Hamburg: Anchor Academic Publishing, 2016).
16. Judi Jennings, *Gender, Religion, and Radicalism in the Long Eighteenth Century: The ‘Ingenious Quaker’ and Her Connections* (Aldershot: Ashgate, 2016); Niki Raga Tantri, ‘English as a Global Language Phenomenon and the Need of Cultural Conceptualizations Awareness in Indonesian ELT’, *IJ-ELTS: International Journal of 1 English Language & Translation Studies*, Vol. 1, No. 1 (2013): 37–49. http://citeseerx.ist.psu.edu/viewdoc/download?doi=10.1.1.673.2047&rep=rep1&type=pdf#page=38; Tri Pujiati, Syihabuddin and Dadang Sudana, ‘Gender, Religion, Cultural Background and Directive Speech Acts Politeness on Medical School Students’, *2nd Internasional Conference on Culture and Language in Southeast Asia (ICCLAS 2018)* (Atlantis Press, 2019). https://doi.org/10.2991/icclas-18.2019.18.
17. Sara Mills, *Gender and Politeness* (New York: Cambridge University Press, 2003).
18. Penelope Brown and Stephen C. Levinson, *Politeness: Some Universals in Language Usage* (New York: Cambridge University Press, 1987); Richard J.Watts, Sachiko Ide, and Konrad Ehlich (eds.), *Politeness in Language: Studies in Its History, Theory and Practice* (Berlin: Mouton de Gruyter, 2008).
19. Shoshana Blum-Kulka, ‘You Don’t Touch Lettuce with Your Fingers: Parental Politeness in Family Discourse’, *Journal of Pragmatics*, Vol. 14, No. 2 (1990): 259–288. https://doi.org/10.1016/0378-2166(90)90083-P; Brian Clancy, ‘You’re Fat. You’ll Eat Them All: Politeness Strategies in Family Discourse’. In *The Pragmatics of Irish* English, ed. Anne Barron and Klaus P. Schneider (Berlin: De Gruyter Mouton, 2005), 177–200; Åsa Brumark, ‘Regulatory Talk and Politeness at the Family Dinner Table’, *Pragmatics*, Vol. 16, No. 2–3 (2006): 171–211. https://doi.org/10.1075/prag.16.2-3.06bru; Aimee E. Miller-Ott and Lynne Kelly, ‘A Politeness Theory Analysis of Cell-Phone Usage in the Presence of Friends’, *Communication Studies*, Vol. 68, No. 2 (2017): 190–207. https://doi.org/10.1080/10510974.2017.1299024; Danette Ifert Johnson, ‘Modal Expressions in Refusals of Friends’ Interpersonal Requests: Politeness and Effectiveness’, *Communication Studies*, Vol. 59, No. 2 (2008): 148–163. https://doi.org/10.1080/10510970802062477.
20. Janet Holmes and Maria Stubbe, *Power and Politeness in the Workplace: A Sociolinguistic Analysis of Talk at Work* (Abingdon: Routledge, 2015).
21. John Lofland, *Polite Protesters: The American Peace Movement of the 1980s* (Syracuse, NY: Syracuse University Press, 1993); Robert Ford, Matthew J. Goodwin, and David Cutts, ‘Strategic Eurosceptics and Polite Xenophobes: Support for the United Kingdom Independence Party (UKIP) in the 2009 European Parliament Elections’, *European Journal of Political Research*, Vol. 51, No. 2 (2012): 204–234. https://doi.org/10.1111/j.1475-6765.2011.01994.x.
22. Alexis P.I. Goh and Peirchyi Lii, ‘Examining Leader–Follower Interactions through the Lens of Chinese Politeness’, *China Report*, Vol. 53, No. 3 (2017): 331–353. https://doi.org/10.1177/0009445517711508.
23. Anonymous, ‘Biden Team Says Trump’s Taunts “Beneath Dignity of The Office”’, *BBC News*, 29 May 2019. https://www.bbc.com/news/world-us-canada-48438038.
24. Holmes and Stubbe, *Power and Politeness in the Workplace*; Stephanie Schnurr, Meredith Marra and Janet Holmes, ‘Impoliteness as a Means of Contesting Power Relations in the Workplace’. In *Impoliteness in Language: Studies on Its Interplay with Power in Theory and Practice*, ed. Derek Bousfield and Miriam A. Locher (Berlin: Walter de Gruyter, 2008), 211–230.
25. David A. Morand, ‘Dominance, Deference, and Egalitarianism in Organizational Interaction: A Sociolinguistic Analysis of Power and Politeness’, *Organization Science*, Vol. 7, No. 5 (1996): 544–556. https://doi.org/10.1287/orsc.7.5.544.
26. Elena Stephan, Nira Liberman, and Yaacov Trope, ‘Politeness and Psychological Distance: A Construal Level Perspective’, *Journal of Personality and Social Psychology*, Vol. 98, No. 2 (2010): 268–280. https://doi.org/10.1037/a0016960.
27. Gabriel Moser and Denis Corroyer, ‘Politeness in the Urban Environment: Is City Life Still Synonymous with Civility?’, *Environment and Behavior*, Vol. 33, No. 5 (2001): 611–625. https://doi.org/10.1177/00139160121973151; Mona Domosh, ‘Those “Gorgeous Incongruities”: Polite Politics and Public Space on the Streets of Nineteenth-Century New York City’, *Annals of the Association of American Geographers*, Vol. 88, No. 2 (1998): 209–226. https://doi.org/10.1111/1467-8306.00091.
28. Joel Feinberg, *Offense to Others: The Moral Limits of the Criminal Law* (Oxford: Oxford University Press, 1985), 10–13.
29. Trystan Edwards, *Good and Bad Manners in Architecture* (2nd edition) (London: Tiranti, 1945).
30. Roff Smith, ‘How Sunlight Reflected Off a Building Can Melt Objects’, *National Geographic*, 5 September 2013. https://www.nationalgeographic.com/news/2013/9/130904-walkie-talkie-building-london-melts-sunlight-physics-science/.
31. Mubeen M. Aslam, ‘Are You Selling the Right Colour? A Cross-Cultural Review of Colour as a Marketing Cue’, *Journal of Marketing Communications*, Vol. 12, No. 1 (2006): 15–30. https://doi.org/10.1080/13527260500247827.
32. Ian Rowe, ‘Civility 2.0: A Comparative Analysis of Incivility in Online Political Discussion’, *Information, Communication and Society*, Vol. 18, No. 2 (2015): 121–138. https://doi.org/10.1080/1369118X.2014.940365.
33. Theodore Caplow, ‘Rule Enforcement Without Visible Means: Christmas Gift Giving in Middletown’, *American Journal of Sociology*, Vol. 89, No. 6 (1984): 1306–1323. https://doi.org/10.1086/228017.
34. Anthony Gray, ‘Bloody Censorship: Swearing and Freedom of Speech’, *Alternative Law Journal*, Vol. 37, No. 1 (2012): 37–40. https://doi.org/10.1177%2F1037969X1203700109.
35. Calhoun, ‘The Virtue of Civility’.
36. SaraMills, ‘Impoliteness in a Cultural Context’, *Journal of Pragmatics*, Vol. 41, No. 5 (2009): 1047–1060. https://doi.org/10.1016/j.pragma.2008.10.014.
37. Amy Irwin, ‘That’s Just Rude: Why Being Polite May Not Be a Universal Concept’, *The Conversation*, 4 April 2018. https://theconversation.com/thats-just-rude-why-being-polite-may-not-be-a-universal-concept-94187; Maria Sifianou and Garcés-Conejos Blitvich, ‘(Im)politeness and Cultural Variation’. In *The Palgrave Handbook of Linguistic (Im)politeness*, ed. Jonathan Culpeper, Michael Haugh and Dániel Z. Kádár (London: Palgrave Macmillan, 2017), 571–599.
38. Feinberg, *Offense to Others*.
39. Example Yuling Pan, Daniel Z. Kadar, *Politeness in Historical and Contemporary Chinese* (London and New York: Bloomsbury, 2011).
40. Sune Laegaard, ‘A Multicultural Social Ethos: Tolerance, Respect or Civility’. In *Diversity in Europe*, ed. Gideon Calder and Emanuela Ceva (Abingdon: Routledge, 2011), 94.
41. Lawrence E. Klein, ‘Liberty, Manners, and Politeness in Early Eighteenth-Century England’. *The Historical Journal*, Vol. 32, No. 3 (1989): 583–605. https://doi.org/10.1017/S0018246X00012437.
42. Jeff E. Nash, ‘Bus Riding: Community of Wheels’, *Urban Life*, Vol. 4, No. 1 (1989): 99–124.
43. Li Wei, ‘The Functions and Use of Greetings’, *Canadian Social Science*, Vol. 6, No. 4 (2010): 56–62.
44. George E. Marcus, W. Russell Neuman and Michael MacKuen, *Affective Intelligence and Political Judgment* (Chicago: University of Chicago Press, 2000).
45. For more on biases and other limitations see work from behavioural economics, e.g. Elinor Ostrom, ‘A Behavioral Approach to the Rational-Choice Theory of Collective Action’, *American Political Science Review*, Vol. 92, No. 1 (1998): 1–22. https://doi.org/10.2307/2585925; Herbert A. Simon, ‘A Behavioral Model of Rational Choice’, *The Quarterly Journal of Economics*, Vol. 69, No. 1 (1955): 99–118. https://doi.org/10.2307/1884852.
46. Daniel Kahneman and Amos Tversky, ‘Prospect Theory: An Analysis of Decision Under Risk’, *Econometrica*, Vol. 47, No. 2 (1979): 263–291. https://doi.org/10.1142/9789814417358_0006; Jonathan Mercer, ‘Prospect Theory and Political Science’, *Annual Review of Political Science*, Vol. 8, No. 1 (2005): 1–21. https://doi.org/10.1146/annurev.polisci.8.082103.104911.
47. Judith Trevino et al., ‘Effect of Biting Before Dipping (Double-Dipping) Chips on the Bacterial Population of the Dipping Solution’, *Journal of Food Safety*, Vol. 29, No. 1 (2009): 37–48. https://doi.org/10.1111/j.1745-4565.2008.00137.x.
48. Johanna Mayer, ‘The Origin Of “The Five-Second Rule”: It has to do with Genghis Khan and Julia Child’, Science Friday, 20 February 2019. https://www.sciencefriday.com/articles/the-origin-of-the-five-second-rule/; Gian De Poloni, ‘Double-Dipping More of a Food Safety Risk Than Invoking the Five-Second Rule, Expert Says’, *ABC News*, 10 January 2019. https://www.abc.net.au/news/2019-01-10/double-dipping-more-risky-than-five-second-rule-expert-says/10695668.
49. Example Pierfrancesco Curzi, ‘Salvini a Falconara. “Elezioni Marche, l'aria è buona. Si va a vincere’, *Il Resto del Carlino*, 22 August 2020. https://www.ilrestodelcarlino.it/elezioni-marche/salvini-a-falconara-1.5437308; Andrea Indini, ‘Il Pd impari le buone maniere’, *Il Giornale*, 1 August 2020. https://www.ilgiornale.it/news/politica/pd-impari-buone-maniere-1880946.html.
50. Example Edyvane, ‘Incivility as Dissent’.
51. For more on US racial labels see Tom W. Smith, ‘Changing Racial Labels: From “Colored” to “Negro” to “Black” to “African American”’, *Public Opinion Quarterly*, Vol. 56, No. 4 (1992): 496–514. https://doi.org/10.1086/269339.
52. Laura Ann Hammersley, ‘Volunteer Tourism: Building Effective Relationships of Understanding’, *Journal of Sustainable Tourism*, Vol. 22, No. 6 (2014): 855–873. https://doi.org/10.1080/09669582.2013.839691.
53. Richard Ming Kirk Tan, ‘The Preparation of Volunteers for Deployment in Emergencies’, *Australian Journal of Emergency Management*, Vol. 21, No. 4 (2006): 54–59. https://search.informit.com.au/documentSummary;dn=413166911892492;res=IELHSS.
54. United States Department of the Army, *The U.S. Army/Marine Corps Counterinsurgency Field Manual: U.S. Army Field Manual No. 3–24: Marine Corps Warfighting Publication No. 3–33.5* (Chicago: University of Chicago Press, 2007).
55. Personal conversation with former extremist and current disengagement specialist with the Free Radicals Project. Melbourne, 1 August 2019.
56. Edyvane, ‘Incivility as Dissent’, 95.
57. Sarah Buss, ‘Appearing Respectful: The Moral Significance of Manners’, *Ethics*, Vol. 109, No. 4 (1999): 795–826. https://doi.org/10.1086/233946.
58. Calhoun, ‘The Virtue of Civility’, 259.
59. Calhoun, ‘The Virtue of Civility’, 260 [emphasis added].
60. Lægaard, ‘A Multicultural Social Ethos’, 86.
61. Richard Boyd, ‘The Value of Civility?’, *Urban Studies*, Vol. 43, No. 5–6 (2006): 871. https://doi.org/10.1080%2F00420980600676105.
62. Shoshana Blum-Kulka, ‘You Don’t Touch Lettuce With Your Fingers: Parental Politeness in Family Discourse’, *Journal of Pragmatics*, Vol. 14, No. 2 (1990): 259–288. https://doi.org/10.1016/0378-2166(90)90083-P.
63. Jennifer A Bunk and Vicki J. Magley, ‘The Role of Appraisals and Emotions in Understanding Experiences of Workplace Incivility’, *Journal of Occupational Health Psychology*, Vol. 18, No. 1 (2013): 87–105. https://doi.org/10.1037/a0030987.
64. Brianna Barker Caza and Lilia M. Cortina, ‘From Insult to Injury: Explaining the Impact of Incivility’, *Basic and Applied Social Psychology*, Vol. 29, No. 4 (2007): 335–350. https://doi.org/10.1080/01973530701665108.
65. John Dixon, Mark Levine and Rob McAuley, ‘Locating Impropriety: Street Drinking, Moral Order, and the Ideological Dilemma of Public Space’, *Political Psychology*, Vol. 27, No. 2 (2006): 187–206. https://doi.org/10.1111/j.1467-9221.2006.00002.x.
66. Benjamin M. Walsh et al., ‘Assessing Workgroup Norms for Civility: The Development of the Civility Norms Questionnaire-Brief’, *Journal of Business and Psychology*, Vol. 27, No. 4 (2012): 407–420. https://doi.org/10.1007/s10869-011-9251-4.
67. Patricia Smokler Lewis and Ann Malecha, ‘The Impact of Workplace Incivility on the Work Environment, Manager Skill, and Productivity’, *JONA: The Journal of Nursing Administration*, Vol. 41, No. 1 (2011): 41–47. https://doi.org/10.1097/nna.0b013e3182002a4c.
68. Lynne M. Andersson and Christine M. Pearson, ‘Tit for Tat? The Spiraling Effect of Incivility in the Workplace’, *Academy of Management Review*, Vol. 24, No. 3 (1999): 452–471. https://doi.org/10.5465/amr.1999.2202131.
69. Christine L. Porath and Amir Erez, ‘Does Rudeness Really Matter? The Effects of Rudeness on Task Performance and Helpfulness, *The Academy of Management Journal*, Vol. 50, No. 5 (2007): 1181–1197. https://doi.org/10.5465/amj.2007.20159919.
70. David Nicolas Hopmann, Rens Vliegenthart and Jürgen Maier, ‘The Effects of Tone, Focus, and Incivility in Election Debates’, *Journal of Elections, Public Opinion and Parties*, Vol. 28, No. 3 (2018): 283–306. https://doi.org/10.1080/17457289.2017.1394310.
71. Deborah Jordan Brooks and John G. Geer, ‘Beyond Negativity: The Effects of Incivility on the Electorate’, *American Journal of Political Science*, Vol. 51, No. 1 (2007): 1–16. https://doi.org/10.1111/j.1540-5907.2007.00233.x.
72. William Spelman, ‘Optimal Targeting of Incivility-Reduction Strategies’, *Journal of Quantitative Criminology*, Vol. 20, No. 1 (2004): 63–88. https://doi.org/10.1023/B:JOQC.0000016700.27654.76.
73. Michael R. Wolf, J. Cherie Strachan and Daniel M. Shea, ‘Incivility and Standing Firm: A Second Layer of Partisan Division, *PS: Political Science and Politics*, Vol. 45, No. 3 (2012): 428–434. https://doi.org/10.1017/S1049096512000509.
74. Maisel, L. Sandy, ‘The Negative Consequences of Uncivil Political Discourse’, *PS: Political Science and Politics,* Vol. 45, No. 3 (2012): 405–411. https://doi.org/10.1017/S1049096512000467; Edyvane, ‘Incivility as Dissent’.
75. Zizi Papacharissi, ‘Democracy Online: Civility, Politeness, and the Democratic Potential of Online Political Discussion Groups’, *New Media & Society*, Vol. 6, No. 2 (2004): 259–283. https://doi.org/10.1177%2F1461444804041444.
76. Paul Chilton, ‘Politeness, Politics and Diplomacy’, *Discourse & Society*, Vol. 1, No. 2 (1990): 201–224. https://doi.org/10.1177%2F0957926590001002005.
77. Joy Hendry, *Wrapping Culture: Politeness, Presentation, and Power in Japan and Other Societies* (New York: Oxford University Press, 1995).
78. Bruno Magalhães, ‘The Politics of Credibility: Assembling Decisions on Asylum Applications in Brazil’, *International Political Sociology*, Vol. 10, No. 2 (2016): 133–149. https://doi.org/10.1177%2F0967010618783640.
79. Erica Chenoweth and Maria J. Stephan, *Why Civil Resistance Works: The Strategic Logic of Nonviolent Conflict* (New York: Columbia University Press, 2011).
80. Oliver Kaplan, *Resisting War: How Communities Protect Themselves* (New York: Cambridge University Press, 2017).
81. Edyvane, ‘The Passion for Civility’; Michael J. Meyer, ‘Liberal Civility and the Civility of Etiquette: Public Ideals and Personal Lives’, *Social Theory and Practice*, Vol. 26, No. 1 (2000): 69–84. https://doi.org/10.5840/soctheorpract20002613.
82. Edyvane, ‘The Passion for Civility’, 345.
83. Bernard E. Harcourt, ‘The Politics of Incivility’, *Arizona Law Review*, Vol. 54, No. 2 (2012): 349 [original emphasis]. https://arizonalawreview.org/pdf/54-2/54arizlrev344.pdf.
84. Edyvane, ‘The Passion for Civility’, 345.
85. Richard Boyd, *Uncivil Society: The Perils of Pluralism and the Making of Modern Liberalism* (Cambridge, MA: Harvard University Press, 1996), 78.
86. Robert Pippin, ‘The Ethical Status of Civility’. In *Civility*, ed. Leroy S. Rouner (Notre Dame, IN: University of Notre Dame Press, 2000), 111–113.
87. Christopher F. Zurn, ‘Political Civility: Another Idealistic Illusion’, *Public Affairs Quarterly*, Vol. 27, No. 4 (2013): 341–368. https://www.jstor.org/stable/43575586.
88. Andrew Peterson, *Civility and Democratic Education* (Singapore: Springer, 2019), 29.
89. Cf. Jeremy Waldron, *The Harm in Hate Speech* (Cambridge, MA: Harvard University Press, 2012).
90. Teresa M. Bejan, 2017. *Mere Civility: Disagreement and the Limits of Toleration* (Cambridge, MA: Harvard University Press), 7.
91. Buss, ‘Appearing Respectful’, 801.
92. Aurelia Bardon, Matteo Bonotti, William Ridge and Steven Zech, ‘Politeness, Public-Mindedness and Their Connection’, unpublished manuscript.
93. Giovanni Tiso, ‘On Polite Nazis and the Violence of Speech’, *Overland*, 6 December 2017. https://overland.org.au/2017/12/the-violence-of-speech/.
94. Edyvane, ‘Incivility as Dissent’.
95. Rawls, *Political Liberalism*.
96. Meyer, ‘Liberal Civility and the Civility of Etiquette’, 72.
97. Rawls, *Political Liberalism*, 224.
98. Jonathan Quong, *Liberalism Without Perfection* (Oxford: Oxford University Press, 2011), 207.
99. Rawls, *Political Liberalism*, 224.
100. Jonathan Quong, ‘The Scope of Public Reason’, *Political Studies*, Vol. 52, No. 2 (2004): 233–250. https://doi.org/10.1111%2Fj.1467-9248.2004.00477.x.
101. Matteo Bonotti, *Partisanship and Political Liberalism in Diverse Societies* (Oxford: Oxford University Press, 2017); Cécile Laborde, *Liberalism’s Religion* (Cambridge, MA: Harvard University Press, 2017).
102. Rawls, *Political Liberalism*.
103. Stephen Macedo, ‘Why Public Reason? Citizens’ Reasons and the Constitution of the Public Sphere’, manuscript available through the Social Science Research Network, published online on 23 August 2010. https://dx.doi.org/10.2139/ssrn.1664085; Quong, *Liberalism Without Perfection*.
104. Gerald Gaus, *The Order of Public Reason: A Theory of Freedom and Morality in a Diverse and Bounded World* (Cambridge: Cambridge University Press, 2011); Kevin Vallier, *Liberalism and Public Faith: Beyond Separation* (New York: Routledge, 2014).
105. Jonathan Quong, ‘Public Reason’. In *The Stanford Encyclopedia of Philosophy*, ed. Edward N. Zalta, 2018. https://plato.stanford.edu/archives/spr2018/entries/public-reason/.
106. John Rawls, *A Theory of Justice* (Cambridge: Harvard University Press, 1999), 153.
107. Jonathan Quong, ‘Cultural Exemptions, Expensive Tastes, and Equal Opportunities’, *Journal of Applied Philosophy*, Vol. 23, No. 1 (2006): 59–60. https://doi.org/10.1111/j.1468-5930.2006.00320.x; Laborde, *Liberalism’s Religion*, 61–62.
108. Steven Wall, *Liberalism, Perfectionism and Restraint* (Cambridge: Cambridge University Press, 1998); Joseph Chan, ‘Legitimacy, Unanimity, and Perfectionism’, *Philosophy and Public Affairs*, Vol. 29, No. 1 (2000): 5–42. https://doi.org/10.1111/j.1088-4963.2000.00005.x.
109. Bejan, *Mere Civility*, 13.
110. Bejan, *Mere Civility*, 14.
111. Waldron, *The Harm in Hate Speech*.
112. For recent discussions of conspiracy theories, see Russell Muirhead and Nancy L. Rosenblum, *A Lot of People Are Saying: The New Conspiracism and the Assault on Democracy* (Princeton: Princeton University Press, 2019); Matej Cíbik and Pavol Hardoš, ‘Conspiracy Theories and Reasonable Pluralism’, *European Journal of Political Theory*, published online on 1 April 2020. https://doi.org/10.1177%2F1474885119899232.
113. Gabriele Badano and Matteo Bonotti, ‘Rescuing Public Reason Liberalism’s Accessibility Requirement’, *Law and Philosophy*, Vol. 39, No. 1: 35–65. https://doi.org/10.1007/s10982-019-09360-8.

